# *Legionella pneumophila *serogroup 3 pneumonia in a patient with low-grade 4 non-Hodgkin lymphoma: a case report

**DOI:** 10.1186/1752-1947-5-387

**Published:** 2011-08-17

**Authors:** Antonella Mencacci, Cristina Corbucci, Alessio Castellani, Paolo Furno, Francesco Bistoni, Anna Vecchiarelli

**Affiliations:** 1 Department of Experimental Medicine and Biochemical Sciences, Microbiology Section, University of Perugia, Via del Giochetto, Perugia 06122, Italy; 2 Department of Clinical and Experimental Medicine, Internal Medicine and Oncology Section, Santa Maria della Misericordia Hospital, Sant'Andrea delle Fratte, Perugia, Italy

## Abstract

**Introduction:**

Nosocomial legionellosis has generally been described in immunodepressed patients, but *Legionella pneumophila *serogroup 3 has rarely been identified as the causative agent.

**Case presentation:**

We report the case of nosocomial *L. pneumophila *serogroup 3 pneumonia in a 70-year-old Caucasian man with non-Hodgkin lymphoma. Diagnosis was carried out by culture and real-time polymerase chain reaction of bronchoalveolar lavage fluid. The results of a urinary antigen test were negative. A hospital environmental investigation revealed that the hospital water system was highly colonized by *L. pneumophila *serogroups 3, 4, and 8. The hospital team involved in the prevention of infections was informed, long-term control measures to reduce the environmental bacterial load were adopted, and clinical monitoring of legionellosis occurrence in high-risk patients was performed. No further cases of *Legionella *pneumonia have been observed so far.

**Conclusions:**

In this report, we describe a case of legionellosis caused by *L. pneumophila *serogroup 3, which is not usually a causative agent of nosocomial infection. Our research confirms the importance of carrying out cultures of respiratory secretions to diagnose legionellosis and highlights the limited value of the urinary antigen test for hospital infections, especially in immunocompromised patients. It also indicates that, to reduce the bacterial load and prevent nosocomial legionellosis, appropriate control measures should be implemented with systematic monitoring of hospital water systems.

## Introduction

Legionnaires' disease is often a hospital-acquired infection. In Italy, 9.2% of nosocomial cases were recorded in 2009 with a fatality rate (34%) significantly higher than community-acquired cases (12%) [1]. This difference is due to the patients' state of immunodeficiency, which is known to be an important risk factor in contracting *Legionella *pneumonia. Often, hospital water systems are colonized by *Legionella *and this contamination is responsible for most cases of hospital-acquired legionellosis [2]. A link between the presence of the bacterium in hospital water systems and nosocomial legionellosis has been reported [3], suggesting that it is necessary to sample hospital water routinely for *Legionella *and make sure that this microorganism is not present in transplant units or other wards with significantly immunosuppressed patients [4]. Sixteen serogroups of *Legionella pneumophila *are known. Serogroup 1 is the most common in clinical and environmental isolates [5], whereas *L. pneumophila *serogroup 3 has rarely been isolated in immunocompromised patients [6].

## Case presentation

In this report, a case of hospital-acquired pulmonary legionellosis in an immunocompromised patient - a 70-year-old Caucasian man - is described. Sixteen years before he presented to us, he had a condition that was diagnosed as low-grade non-Hodgkin lymphoma of the B-cell small lymphocytic type (stage IV with mediastinal, abdominal, bone marrow, and superficial node site involvement), and nine courses of combined polychemotherapy were administered with cyclophosphamide, doxorubicin, vincristine, and prednisone. Four years before he presented to us, lymphoma chemotherapy was started again with cyclophosphamide and fludarabine because of a progression of mesenteric involvement of the disease. This regimen was interrupted four months later because of toxicity, and 11 courses of alemtuzumab were administered thereafter in the Oncology and Hematology Clinic of our division.

Three years before he presented to us, he was discharged from our ward after a two-week hospitalization for fever and pancytopenia (first hospital admission of this report). After seven days, he was hospitalized again, in another ward of the same hospital, because of fever, dyspnea, and bowel movements with loose stools (second admission). A chest X-ray showed an inflammatory infiltrate at the base of his right lung, and another slight infiltration seemed to be spreading in the upper field of his left lung. The results of laboratory tests were unremarkable except for significant increases of erythrocyte sedimentation rate and C-reactive protein, minimal increases of aspartate aminotransferase and alanine aminotransferase, and mild anemia. The results of blood cultures, a cytomegalovirus antigen test in serum, and a *Clostridium difficile *toxin stool test were negative. A course of empirical antibiotic therapy with piperacillin/tazobactam plus ciprofloxacin was started. A rapid defervescence ensued, and our patient was discharged after an eight-day course of antibiotics. However, two days after discharge, fever recurred and he was hospitalized again in our ward to investigate a possible opportunistic respiratory infection (third admission). He appeared mildly ill and complained of pleuritic pain at the base of his right lung when breathing deeply; a low-grade fever was present, but his respiratory rate (20 breaths per minute), heart rate (88 beats per minute), and blood pressure (120/70 mm Hg) were normal; his arterial blood oxygen saturation was 96% while he was breathing room air. Inspiratory crackles were evident over his right lower lung field, but the results of the physical examination were otherwise normal. Blood cultures were requested in order to check for common pathogens as an empiric antibiotic therapy was started with piperacillin/tazobactam. However, several hours after admission, his fever increased to over 38°C and persisted for five days. Therefore, a chest computed tomography (CT) scan and a bronchoscopy test with bronchoalveolar lavage (BAL) were performed and, although our patient's general clinical condition remained stable and blood culture results were negative, beta-lactams were replaced with clarithromycin to provide antibacterial coverage against possible atypical respiratory pathogens. The chest CT scan showed evidence of an area of lung parenchymal consolidation in his right lower lobe with adjacent ground-glass opacities and micronodules. No lymphadenopathy was evident. A microbiological examination of BAL fluid was negative for common Gram-negative and Gram-positive pathogens, fungi, *Nocardia *spp., *Mycobacterium tubercolosis, Pneumocystis jiroveci*, cytomegalovirus, adenovirus, and respiratory syncytial virus, but *Legionella *was detected after four days of incubation. For culture, BAL fluid, undiluted or diluted (1:10) in trypticase soy broth, was plated on a buffered charcoal yeast extract (BCYE) medium with 0.1% alpha-ketoglutaric acid and on selective BCYE agar medium supplemented with glycine, vancomycin, polymyxin B, and cycloheximide (GVPC) (Becton Dickinson, Milan, Italy). Colonies were identified as *L. pneumophila *serogroup 3 by using the Dresden monoclonal antibody panel [7]. The results of real-time polymerase chain reaction (PCR) assays performed on DNA extracted from BAL fluid were also positive for *Legionella *spp. (Nanogen Advanced Diagnostic, Torino, Italy). The results of urinary antigen tests for *L. pneumophila *serogroup 1 (Binax, Portland, ME, USA) and non-serogroup 1 (Biotest, Dreieich, Germany) were negative.

A prompt clinical response was observed after clarithromycin treatment. Two days after the start of macrolide treatment, our patient was afebrile and had no chest pain and his condition was generally improving and remained so thereafter. A chest X-ray performed two weeks later showed a slight reduction of right lung opacity, and he was discharged after 20 days of hospital stay while on clarithromycin therapy. This treatment was continued for seven more days. A follow-up chest CT scan obtained five weeks later revealed an almost complete resolution of the previously documented right lung consolidation and ground-glass areas.

An environmental investigation was carried out after the isolation of the infectious agent. First of all, our patient's home water system was investigated for the presence of *L. pneumophila*, but this pathogen was found in neither the cold nor the hot water system (data not shown). Given that the incubation time for *L. pneumophila *is generally two to 10 days and that, six days after being discharged from his first hospitalization (for pancytopenia), our patient was admitted again with respiratory symptoms, we suspected that he had been exposed to the microorganism during the period spent in the hospital. To verify this hypothesis, an intensive environmental investigation was carried out on the hospital's hot and cold water systems. A microbiological analysis of water samples, collected from the sink and shower of our patient's room from the first hospital admission, revealed that the hot water sample was contaminated by *L. pneumophila *serogroup 3 (Table 1). The concentration of 8000 colony-forming units per liter (CFU/L) exceeded the 1000 CFU/L of the European guidelines for hotels and the 1000 CFU/L of the Italian guidelines for hospitals [8]. Also, a lot of other sites of the hospital's hot water system were highly colonized by *L. pneumophila*, including serogroups 3, 4, and 8 (Table 2). Legionella was not isolated from the cold water system. Molecular typing of *L. pneumophila *clinical and environmental isolates was carried out by using the amplified fragment length polymorphism method [9], and the genomic profile of the *L. pneumophila *clinical strain matched with that of the *L. pneumophila *serogroup 3 strain (data not shown).

**Table 1:** Microbiological analysis of hot water sampled from our patient's room from his first admission

Sampling site	Sampling mode	*Legionella pneumophila *load, CFU/L
Bathroom sink	Pre-flushing	4 × 10^3^
	Post-flushing	4.5 × 10^3^
Shower	Pre-flushing	4 × 10^2^
	Post-flushing	3.5 × 10^2^

CFU/L: colony-forming units per liter.

**Table 2:** Microbiological analysis of hot water sampled from the entire water system of the hospital

Sampling site	Sampling mode	*Legionella pneumophila *load, CFU/L
Water mains	Pre-flushing	Not detected
	Post-flushing	Not detected
Hospital water harvesting tanks	Pre-flushing	Not detected
	Post-flushing	Not detected
Collector pipes	Pre-flushing	Not detected
	Post-flushing	Not detected
Boiler	Pre-flushing	5 × 10^3^
	Post-flushing	4.5 × 10^3^
Room closest to the boiler (sink)	Pre-flushing	6.5 × 10^3^
	Post-flushing	7 × 10^3^
Room farthest from the boiler (sink)	Pre-flushing	4 × 10^3^
	Post-flushing	5 × 10^2^

CFU/L: colony-forming units per liter.

On the basis of these results, an extensive program of microbiological controls in the entire hospital water system was implemented. In accordance with the European guidelines, shock hyperchlorination was applied to the water distribution system [10] with a single addition of chlorine to the water to obtain concentrations of free residual chlorine of 20 to 50 mg/L throughout the water system, including the distal point. After this treatment, cultures of water samples collected one day after hyperchlorination showed a decrease in *L. pneumophila *concentration. However, a considerable increase of CFU was observed one month later (Table 3). Therefore, continuous hyperchlorination treatment (free residual chlorine of 1 to 3 mg/L) of the hospital water system is now routinely applied and monitoring is performed every six months. In addition, periodic monitoring of the *Legionella *CFU in the water system is carried out along with careful clinical surveillance of legionellosis cases in low- and high-risk patients with pneumonia. No other nosocomial *Legionella *pneumonia cases have been observed so far.

**Table 3:** Results of microbiological analysis from the hospital's hot water samples one day and one month after hyperchlorination

*Legionella pneumophila *load, CFU/L				
**Sampling site**	**Sampling mode**	**Before treatment**	**One day after treatment**	**One month after treatment**

Boiler	Pre-flushing	5 × 10^3^	1.5 × 10^2^	3.5 × 10^3^
	Post-flushing	4.5 × 10^3^	2 × 10^2^	5 × 10^3^
Room closest to the boiler (sink)	Pre-flushing	6.5 × 10^3^	4 × 10^2^	8.5 × 10^3^
	Post- flushing	7 × 10^3^	2.5 × 10^3^	6 × 10^3^
Room farthest from the boiler (sink)	Pre-flushing	4 × 10^3^	2.5 × 10^1^	2.5 × 10^3^
	Post-flushing	5 × 10^2^	1.5 × 10^2^	3.5 × 10^2^
Patient's room from first admission (sink)	Pre-flushing	4 × 10^3^	2 × 10^2^	2.5 × 10^3^
	Post-flushing	4.5 × 10^3^	4 × 10^2^	3 × 10^3^

CFU/L: colony-forming units per liter.

## Discussion

Owing to the difficulty of distinguishing legionellosis from other forms of pneumonia by clinical and roentgenographic analysis, specific laboratory diagnostics should be enforced in order to increase the detection rate of nosocomial Legionnaires' disease. Culture remains the most specific diagnostic procedure for legionellosis [5]. The usefulness of urinary antigen detection for the diagnosis of Legionnaires' disease is well documented. However, because it is fast and easy to perform, this test has caused a decrease in the use of cultures to detect infection, resulting in incomplete surveillance for legionellosis [5]. The Binax urinary antigen kit detects *L. pneumophila *serogroup 1 antigen [11], whereas the Biotest urine antigen enzyme immunoassay has a wide range of cross-reactivity to the other serogroups and species. Benson and colleagues [12] reported that both Binax and Biotest urinary antigen kits were capable of detecting multiple serogroups of *L. pneumophila*, including serogroup 3. However, in this case and in other legionellosis cases [6], the urine antigen test was negative when the above-mentioned kits were used and the correct diagnosis was made only when BAL fluid-specific culture and real-time PCR methods were used. This demonstrates the importance of using at least one of these methods as part of the routine microbiological testing of BAL fluid, especially in immunocompromised patients with pulmonary infiltrates. In addition, every time that the clinical features are equivocal, a negative result in urinary antigen tests should not be a reason for ruling out the disease.

Our patient was first empirically treated with clarithromycin to cover possible intracellular atypical respiratory pathogens [13]. After the isolation of the infectious agent, this treatment was not replaced with other antibiotics such as levofloxacin [14], because of the prompt clinical response to this macrolide and because of the previous poor response to a regimen with an antibiotic containing fluoroquinolone (ciprofloxacin). Indeed, the use of a fluoroquinole (ciprofloxacin) not specific for pulmonary infections and the short course (eight days) of treatment in a severely immunosuppressed patient could concur with the recurrence of respiratory symptoms and could have induced the third hospitalization.

Colonization of water systems by *Legionella *spp. occurs in hospitals throughout the world [15]. In hospital wards where immunosuppressed patients are subjected to chemotherapy drugs, hot water systems should be free of *Legionella *contamination.

The water system of our hospital was colonized by *L. pneumophila *of different serogroups. Although various methods to control *Legionella *in water distribution systems have been described in the literature (for example, methods based on physical or chemical treatments or both), none of these treatments is able to eradicate the bacterium permanently, and re-colonization occurs as soon as the treatments are interrupted [16]. Also, in our experience, the ineffectiveness of hyperchlorination treatments in eradicating *L. pneumophila *was demonstrated by the re-growth of *Legionella *only one month later to levels similar to those observed before treatment. This suggests a need for continuous monitoring and a maintenance regime for the hospital plumbing system, particularly in wards accommodating high-risk patients.

## Conclusions

This case report demonstrates that (a) culture of respiratory secretions and real-time PCR for *L. pneumophila *should be part of the routine microbiological testing of BAL fluid from immunocompromised patients with pulmonary infiltrates, (b) a negative result from a urinary antigen test does not rule out the presence of legionellosis, and (c) the monitoring of the *Legionella *species in hospital water systems can help to bring in control measures to reduce the bacterial load and is therefore a proactive strategy for the control of the *Legionella *infection in hospitalized patients.

## Abbreviations

BAL: bronchoalveolar lavage; BCYE: buffered charcoal yeast extract; CFU: colony-forming units; CT: computed tomography; PCR: polymerase chain reaction.

## Consent

Written informed consent was obtained from the patient for publication of this case report and any accompanying images. A copy of the written consent is available for review by the Editor-in-Chief of this journal. The admitting hospital approved the use of patient samples and data.

## Competing interests

The authors declare that they have no competing interests.

## Authors' contributions

CC carried out the environmental investigation. AM performed the microbiological diagnosis and drafted the manuscript. AC and PF were responsible for the clinical management and therapy. AV drafted the manuscript. FB helped to draft the manuscript. All authors read and approved the final manuscript.
